# Prayer as a Medicine

**Published:** 1875-09

**Authors:** 


Prayer as Medicine.—The newspapers say that a religious sect, assuming the appropriate designation of the "Peculiar People," has established a hospital in London for the treatment of disease by prayer exclusively. The theory is that disease is a visitation of God, and not to be interfered with by outside agencies of human contrivance. There is nothing new in the doctrine. It was the prevalent doctrine in the dark ages of antiquity, when a heathen priesthood monopolized the treatment of disease, and when incantations and orgies of various kinds constituted the materia medica. We do not doubt that the hospital of the "Peculiar People" will make a good showing of success. The large majority of their patients would get well if left to nature alone, without the aid even of prayer; and *with* the hope and confidence inspired by faith in that instrumentality, many will recover who might die if left to nature alone. With the innate tendency of the living organism to cast off disease and restore healthy action, it is impossible to determine precisely the parts performed respectively by nature and by medicine, in the curative process. Not even the professional mind, schooled to observation on such subjects, can make the distinction, except in a limited proportion of cases. For this reason the history of medicine is a history of delusions, not only as to the popular mind, but to a great extent among professional observers. No theory, no conceit, no superstition, no remedy, but has had its believers, its successes and its triumphs. Many times the reputed success and triumph of treatment over disease is in reality a triumph of nature over both disease and treatment. Life is not easily destroyed by such agencies as are employed with the design of curing disease. How many women are there at any given time in any civilized community conspiring against the young life in the matrix, and doing their worst, by drugs and by violence, to destroy its vitality? And when the inherent *vis* of the germ is capable of protecting it from such rude assaults, and even forcing its growth all the while, what wonder is it that the same preservative energy carries the fully developed being safely through the shock of violence or of unwise medication? And to return to the subject of prayer as medicine, if people will get well without it, why not with it? Nay, let us ask, would not a very large proportion of the patients of the "Peculiar People's" hospital recover if the whole current of prayer were offered up for their death instead of their recovery?—*Pacific Med. and Surg. Jour.*

